# Incidence of Spinal Perineurial (Tarlov) Cysts among East-European Patients

**DOI:** 10.1371/journal.pone.0071514

**Published:** 2013-08-01

**Authors:** Franciszek Burdan, Agnieszka Mocarska, Marzena Janczarek, Robert Klepacz, Marek Łosicki, Krzysztof Patyra, Agnieszka Brodzik, Joanna Kiszka, Aneta Chruścicka, Iwonna Żelzowska-Cieślińska, Elżbieta Starosławska

**Affiliations:** 1 Department of Radiology, St. John’s Cancer Center, Lublin, Poland; 2 Department of Human Anatomy, Medical University of Lublin, Lublin, Poland; 3 Department of Neuroradiology and Interventional Radiology, Medical University of Lublin, Lublin, Poland; 4 Department of Clinical Pathology, Medical University of Lublin, Lublin, Poland; 5 Department of Radiotherapy, St. John’s Cancer Center, Lublin, Poland; 6 Department of Clinical Oncology, St. John’s Cancer Center, Lublin, Poland; UCLA, United States of America

## Abstract

The spinal perineurial cyst (Tarlov) is a dilatation between the perineurium and endoneurium of spinal nerve roots, located at level of the spinal ganglion and filled with cerebrospinal fluid but without communication with the perineurial subarachnoid space. The aim of the study was to evaluate it incidence among East-European patients. The retrospective data collected during various magnetic resonance spinal examinations and stored on the picture archiving and communication system was analyzed for an incidence of perineurial cysts. From among 842 patients that underwent examination, 75 cases perineurial cysts were revealed. In 22 cases single anomalies were found. In remaining 53 cases, multiple uni- or less frequently bilateral changes were noted. The most common position was the sacral canal, particularly the level of S2 and S3. Occasionally, cysts were also visible on the cervical, thoracic and lumbar level. Incidence of sacral perineurial cysts was significantly higher in females than in males. Similar data was found for single and multiple changes despite of their localization. Insignificant changes were seen for patient age and cyst size. Perineurial spinal cysts were the most frequently observed on the sacral level and such changes were more common in females.

## Introduction

The spinal perineurial cyst is a rare anomaly of the nervous system. It is also known as Tarlov cyst, since was described for the first time by Isadore M. Tarlov (1905–1970) in 1938. The pathology is defined as a cystic dilatation between the perineurium and endoneurium of spinal nerve roots, located at level of the spinal ganglion and filled with cerebrospinal fluid but without communication with the perineurial subarachnoid space [1]–[8]. The neuronal fibers are always present inside or on the wall of the cyst that is usually formed by the perineurium, neuronal (peripheral nerve fibers and ganglionic cells) and fibrous connective tissue [2], [8]. They could be seen as a single or multiple lesions on various levels but the most common position is S1–S4. The cyst belongs to type II of meningeal cysts – according to Nabors et al. [4], which also contains so-called meningeal diverticuli that are positioned proximally to the spinal ganglion and communicate with the subarachnoid space. Most of such changes are observed on the thoracic level, less frequently on the lumbo-sacral and cervical one. The other two types of meningeal cyst are extra- and intradural changes. Unlike perineurial, extradural cysts (type I) are not connected with roots or any other parts of spinal nerves. They are usually congenital abnormalities located in the sacral canal as intra-sacral meningoceles, frequently associated with other developmental changes. Their enlarged part is connected by a narrow opening/canal with a dural sac. The third type includes intradural cysts but their localization deeply depends on etiology. Congenital changes are reported generally posteriorly to the spinal cord, while posttraumatic ones in front of the organ [4]. According to the classical Tarlov’s description the prolongation of subarachnoid space should be also pointed [8].

The aim of the study was to evaluate an incidence of the spinal perineurial cysts among East-European patients that underwent magnetic resonance (MR) examination.

## Materials and Methods

The entire study was conducted on the retrospective data collected during magnetic resonance diagnostic examinations in Radiology Department of St. John’s Cancer Center (Lublin, Poland), since January 1, 2011 until December 31, 2012. The protocol was fully approved by Institutional Review Board (St. John's Cancer Center; Lublin, Poland), but according to the national law no written permission was obtained from patients, since personal data was not presented and analyzed throughout the study and in the final report. In Poland, no written permission from patients is needed to evaluated and used any data obtained during various diagnostic examinations and/or standard therapeutic procedures for any retrospective scientific analysis, as long as patient name, ethnicity, sexuality, religion, etc. will not be pointed in the final report. Such regulation could be downloaded directly from official web-side of Polish Parliament (http://isap.sejm.gov.pl/DetailsServlet?id=WDU19971330883 - Journal of Law 1997 No. 133/883, article 27, section 2.9).

All studies were performed on 1.5T MR scanner (Achieva; Philips Medical Systems; Veenpluis, The Netherlands) according to the standard protocol for the cervical, thoracic and lumbar spinal imagining, which included routine sagittal and axial series. The field of view varied depends on examined area, while section thickness and intersection gap was constant and kept at 3 and 0.3 mm, respectively in case of the cervical (C), thoracic (T), thoraco-cervical (TC) and thoraco-lumbar (TL) part of the vertebral column. For the lumbo-sacral (LS) part both values were kept at 4 and 0.4 mm, respectively. T1- (repetition time 500–1500 ms) and T2-weighted (repetition time 3000–4000 ms) turbo and fast spin echo, with and without fat suppression (FS) were performed (flip angle 90^o^). Additional coronal scans and other sequences, as well as gadolinium-diethylene-triamine-penta-acetic acid (Gd-DTPA) injections were used on the request of the supervising radiologist. Data was stored on the picture archiving and communication system (PACS) and examined by radiologists who had at lest 10 years of clinical experience.

All the positive findings (in diameter over 1 mm) were evaluated in relation to their number, anatomical level, size, patient age and gender. Data was presented as the absolute and relative value, in relation to the number of all patients examined in the proper part of vertebral column. Based on classical anatomical and radiological description, the spine was divided into cervical, thoracic, lumbar and sacral (lumbo-sacral) parts and cysts were named according to the associated spinal nerve, i.e. above vertebral body on the cervical level (e.g. cyst on the C5 level was visible in the intervertebral foramen between C4 and C5 vertebrae) or below vertebral body in the remaining part of vertebral column (e.g. cyst on the T5 level was visible in the intervertebral foramen between T5 and T6 vertebrae). In case patient underwent simultaneous examination of two parts (cervical and thoracic; thoracic and lumbar, lumbar and sacral) such data was used for both proper regions, just to calculate the total number of MR examinations. Data from multiple examinations of the same region, performed in one patient, was limited only to the first or the most representative one. However, in case of patient that underwent examination of different regions of the vertebral column such data was incorporated to each proper area of the spine.

The largest sagittal diameter of each cyst was measured using automatic tool of MR working station (Philips Extended MR Work Space 2.6.3.4; Philips Medical Systems; Veenpluis, The Netherlands). In case of multiple changes, only measurement of the biggest lesion was taken for the final statistical analysis.

The distribution of continuous data was analyzed by Kolmogorov–Smirnov test, and differences were evaluated by Mann–Whitney *U*-test. The nominal scale measures were analyzed by *χ* ^2^ Fischer exact test. The 0.05 level of probability (*p*<0.05) was used as the criterion of significance.

## Results

**Figure 1:**
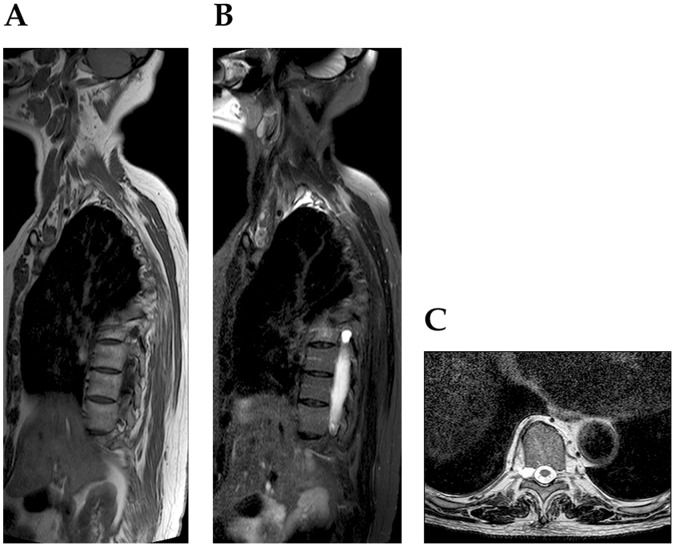
60-year-old woman with a perineurial cyst in the right T6 intervertebral foramen (between T6-T7), other cysts are not visible due to scoliosis. Sagittal section on T1- (A) and T2-weighted (B) images. Axial T2-weighted (C) image.

**Figure 2:**
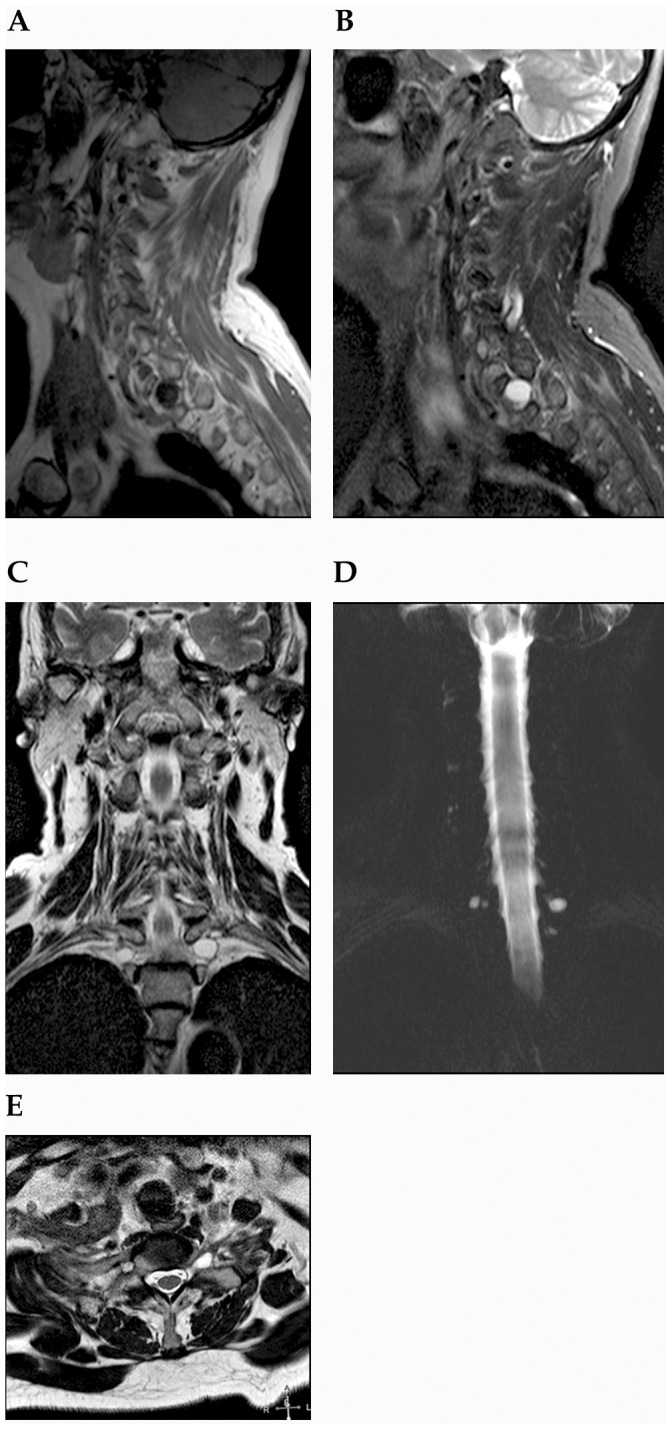
63-year-old woman with bilateral perineurial cysts on the T1 level (between T1 and T2). Sagittal section on T1- (A) and FST2-weighted (B) images with a view of the left lesion. Frontal T2-weighted section (C) and MR mielography (D). Axial T2-weighted (E) image.

**Figure 3:**
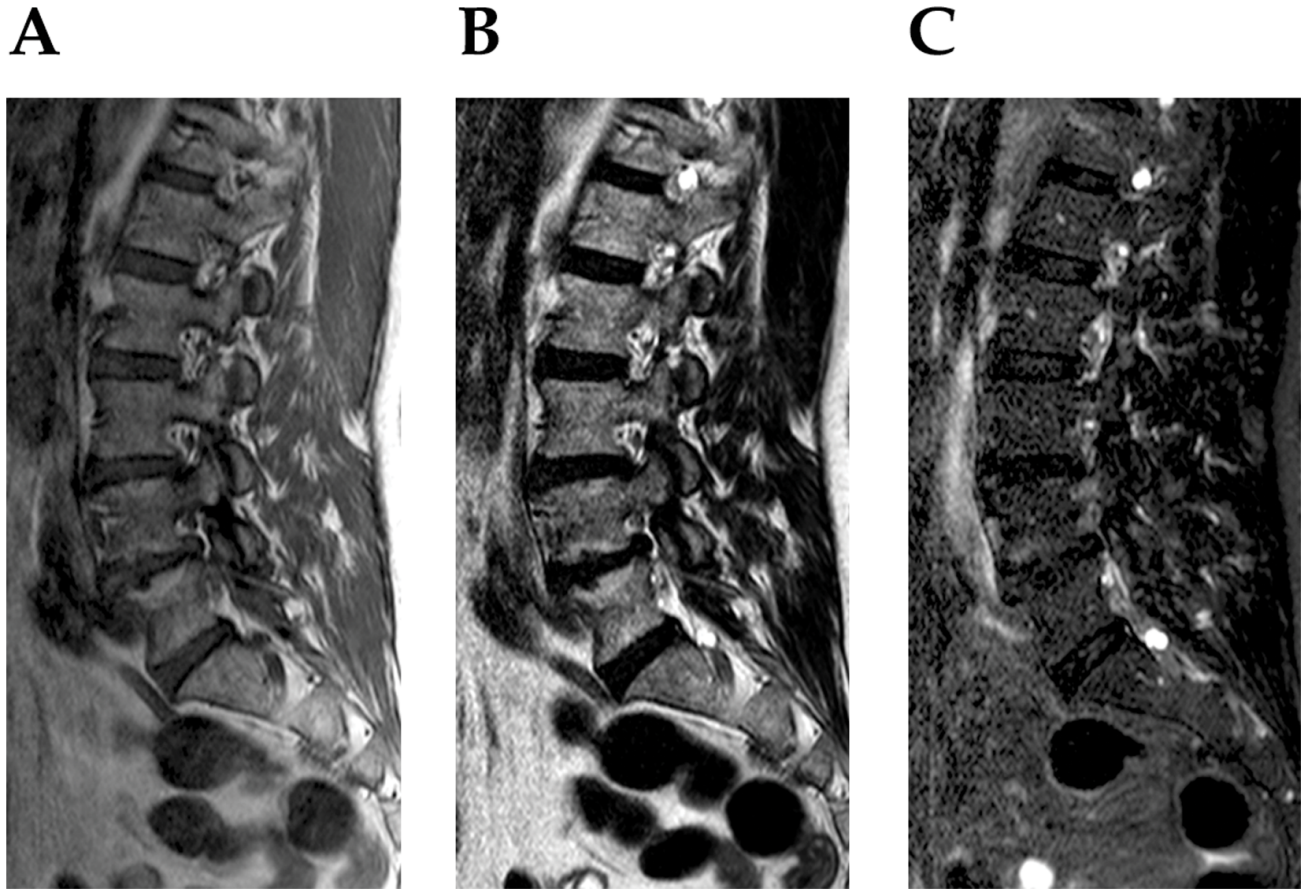
65-year-old woman with right side perineurial cysts on the T12 (between T12 and L1) and L5 level (between L5 and S1). Sagittal section on T1- (A), T2- (B) and FST2-weighted (C) images.

All reported perineurial spinal cysts were characterized by a classic radiological morphology. They were visible on the level of intervertebral foramens or inside of the sacral canal as an oval or circular uniform changes hyperintense on T2- and hypointense (or intermediate) on T1-weighted sequences (fig. 1–3). Their signal corresponded to the cerebrospinal fluid inside of the subarachnoid space that surrounds the spinal cord or cauda equina. The thin rim of signal void was always found around them. In case of Gd-DTPA injections, no contrast enhancement of the cyst was found.

From among 842 patients that underwent magnetic resonance examination of various spinal regions in the selected period, 75 cases of perineurial cysts were revealed (Table 1). Only in 22 cases single anomalies were found. In remaining 53 cases, multiple uni- or less frequently, bilateral changes were observed (fig. 1–3). The most common position was the sacral canal, particularly the level of S2 and S3 (Table 1–2). Occasionally, cysts were also visible on cervical, thoracic and lumbar level. Interestingly, all cervical lesions were seen in females. An incidence of perineurial cysts was significantly higher in females than in males on the sacral level (Table 1). Similar data was found for single and multiple changes despite of their localization. Such observations were proved for separately evaluated years, as well as for the total data.

**Table 1:** Incidence of perineurial cysts in relation to the vertebral/spinal level.

Spinal level	2011				2012				Total			
	Female		Male		Female		Male		Female		Male	
	n^a^	%^b^	n^a^	%^b^	n^a^	%^b^	n^a^	%^b^	n^a^	%^b^	n^a^	%^b^
Cervical^c^	2/62	3.23	0/43	0.00	1/109	0.92	0/43	0.00	3/171	1.75	0/86	0.00
Thoracic^c^	4/44	9.09	1/17	5.88	5/85	5.88	1/53	1.89	9/129	6.98	2/70	2.86
Lumbar^c^	1/149	0.67	0/80	0.00	1/154	0.65	3/94	3.19	2/303	0.66	3/174	1.72
Sacral^c^	28/146	19.18*	5/74	6.76	24/122	19.67*	7/80	8.75	52/268	19.40*	12/154	7.79

Absolute number of MR examinations per selected spinal region: cervical –221 (146/75; female/male), thoracic –108 (69/39), thoraco-cervical –36 (25/11), thoraco-lumbar –55 (35/20), lumbo-sacral –422 (268/154).

a absolute number of perineurial cysts/total number of examinations in which the proper part of vertebral column was evaluated.

b relative incidence (%) of perineurial cysts in relation to the total number of the proper level of vertebral column.

c each patient may be seen more then once in table since some multiple cysts were seen on adjusted parts of the vertebral column (e.g., cervical and thoracic, thoracic and lumbar, lumbar and sacral).

* p<0.05 female vs male.

**Table 2:** Incidence of perineurial cysts depends on their number and localization.

Spinal level	n	%
S2	22	29.33
S2 S3	20	26.66
S1	4	5.33
S1 S2	4	5.33
C7 T1	3	4.00
S1 S2 S3	2	2.67
S3	2	2.67
T1 Th4 T6 T7 T8	1	1.33
T1	1	1.33
T1 T2	1	1.33
T5	1	1.33
T10 T11 T12	1	1.33
T11 T12	1	1.33
T11 T12 S2	1	1.33
T12 L5	1	1.33
L1 L2 L5 S2	1	1.33
L1 S1 S2	1	1.33
L1 S2	1	1.33
L2 L3	1	1.33
L4 L5 S2	1	1.33
L4 S2 S3	1	1.33
S1 S3	1	1.33
S1 S2 S3 S4	1	1.33
S2 S3 S4	1	1.33
S3 S4	1	1.33

Lack of significant differences in age were found between males and females with the cyst. Insignificant changes were also revealed for the maximal diameter of the lesion between the ganders (Table 3). Moreover, no differences were noted for such parameters between single and multiple cysts.

**Table 3:** Age (year) and maximal sagittal diameter (mm) of perineurial cysts or the biggest one in case of multiple changes. Data presented as Mean ± S.D.

	Age	Sagittal diameter
Female	54.80±12.43	11.21±4.02
Male	54.00±14.44	12.50±4.20
Single cysts	56.05±13.35	11.70±4.42
Multiple cyst	54.08±12.55	10.86±3.03

## Discussion

The obtained data shows higher incidence of perineurial cysts on the sacral level than reported in an available literature. Generally, they are classified as rare lesions, since were previously seen only in 1–5% of examined patients [2], [6], [7]. Usually, such pathologies are reported as incidental findings, defined as asymptomatic changes found during examination of a patient for unrelated reasons [6]. Presently, there is growing number of such pathologies, including anatomical variations, due to increase availability of computer tomography and magnetic resonance examinations [6], [9], [10]. In a recently published study, Park et al. [6] reported only 41 cases (3.2%) of anomaly among 1268 patients that underwent magnetic resonance of the lumbar spine but no sex differences were found. Lack of significant sex prevalence (16 out of 27 patients –59% females) was also pointed out by Lucantoni et al. [2]. However, similar to our results, female were more commonly affected (38 out of 54 patients –70% females) in study reported by Longdown et al. [1]. Higher differences were observed among patients with symptomatic cysts. Based on the cumulative analysis done by Oaklander [5] with data presented by other authors [1], [7], [11]–[14], the cyst was found in 29 females (78%) out of 37 patients. Higher female predominance was also confirmed by neurosurgical data since 102 (84%) out 122 patients who underwent precutanous sealing of perineurial cysts were females [3].

All symptoms of perineurial cysts depend on their localization and size. However, most of them are asymptomatic but only about 1% of patients may present various clinical signs [7], [15], in particular sensory disturbances, motor deficits, and dysfunction related to autonomic system. In case of the most common sacral cyst symptoms include: radicular pain, sensory abnormalities and motor weakness on the level of lower limb or lesser pelvis, as well as impotence, urinary and bowel dysfunctions. Symptoms are time-dependent since cysts have tendency to increase size over time and large changes may even modulate morphology of surrounding bones. Acceleration of clinical signs could be also seen in case of an increase pressure of the cerebrospinal fluid, e.g., during coughing, Vasalva maneuver, climbing and in long-time vertical position [15]. Due to lack of proper neurological data in some cases we were not able to state if the reported perineurial cysts were symptomatic. Generally, in most patients various intervertebral disk changes, or less frequently intra- and extraspinal abnormalities were revealed (data not shown).

The main limitation of the study is a relatively small group of examined patients and for this reason a follow-up and mulicenter studies are desirable to prove or to lowered a high incidence of the perineurial cyst among East-European population. Although the study was performed at the oncological hospital the final data – related to incidence and sex-dependencies – should not be deeply influenced by any possible malignancy since most of them develop as congenital changes [4], [7]. However, according to some other hypothesis the evaluated cysts are secondary to a local neuronal inflammation, arachnoid proliferation or develop as a posttraumatic breakage of venous drainage in peri- and epineurium due to the hemosiderin deposition [1]–[4]. It has to be also pointed out that based on radiological data, without histological evaluation, it is not always possible to distinguish perineurial spinal cysts from other meningeal cysts, since – typical for Tarlov cysts – neuronal fibers may not be clearly visible [1], [3].

In conclusion, perineurial spinal cysts were the most frequently observed on the sacral level and such changes were more common in females.
